# Surfactant protein-D (SP-D) gene polymorphisms and serum level as predictors of susceptibility and prognosis of acute kidney injury in the Chinese population

**DOI:** 10.1186/s12882-017-0485-x

**Published:** 2017-02-17

**Authors:** Jiao Liu, Guang Li, Lianghai Li, Zhiyong Liu, Qingshan Zhou, Guirong Wang, Dechang Chen

**Affiliations:** 1 Department of Critical Care Medicine, Renmin Hospital of Wuhan University, Wuhan, Hubei 430060 China; 2 Department of Critical Care Medicine, Jingzhou Central Hospital, Jingzhou, Hubei 434020 China; 3 Department of Internal medicine, Renmin Hospital of Wuhan University, Wuhan, Hubei 430060 China; 4 Department of Surgery, SUNY Upstate Medical University, Syracuse, NY 13210 USA; 5 Department of Critical Care Medicine, Shanghai Ruijin Hospital Affiliated with Jiaotong University, Shanghai, China

**Keywords:** Acute kidney injury, Surfactant protein D, Polymorphism, Kidney injury molecule-1, Susceptibility, Prognosis

## Abstract

**Background:**

Injury to the kidney epithelial barrier is a characteristic feature of acute kidney injury (AKI). Serum surfactant protein-D (SP-D), a known biomarker of damaged alveolar epithelium, is also secreted by renal tubular epithelial cells. Therefore, the aim of this study was to examine the possible association of SP-D with AKI susceptibility and prognosis.

**Methods:**

In this study, 159 AKI patients and 120 healthy individuals were included. SP-D polymorphisms Thr11Met and Thr160Ala, AKI patient serum SP-D levels at days 1, 3 and 7 and urine KIM-1 levels in both AKI patients and controls were examined. The obtained results were correlated with the AKI stage, duration of renal replacement therapy (RRT) and prognosis.

**Results:**

Serum SP-D level in AKI patients was higher than controls (*p* < 0.01). SP-D 11Thr/Thr genotype was more frequent in AKI patients than in controls (*p* < 0.01). Furthermore, AKI patients with SP-D 11Thr/Thr genotype had significantly higher serum SP-D levels (*p* < 0.05) compared to other genotypes. Serum SP-D levels corrected to the progression of AKI with a peak at day 3. Furthermore, the SP-D 11Thr/Thr genotype frequency and baseline serum SP-D level were higher in patients who subsequently died. Baseline serum SP-D levels positively correlated with the urine KIM-1 levels, AKI stage and RRT duration.

**Conclusion:**

In our study, elevated serum SP-D was associated with worse AKI clinical outcomes and patients with SP-D 11Thr/Thr genotype were more susceptible to AKI. Collectively, these findings suggest that SP-D may be useful as a biomarker of AKI susceptibility and prognosis.

## Background

In recent years, the recognition of acute kidney injury (AKI) has increased dramatically worldwide [1]. The incidence rates of hospital-acquired and intensive care unit (ICU)-acquired AKI are approximately 21.6%, and 40%, respectively [2]. The mortality of ICU patients with AKI is 1.5-2 times higher compared to ICU patients without AKI, indicating that AKI can act as an independent risk factor of death in the ICU [2]. At present, risk of development and severity of AKI cannot be reliably predicted from common clinical risk factors. The possible genetic predisposition to AKI or the influence of a certain genetic background on AKI patient outcome still remains to be elucidated [3].

Surfactant protein D (SP-D) is a member of the C-type lectin family and expression and secretion were initially described in lung alveolar epithelial type II cells [4]. In addition to its role in surfactant homeostasis, SP-D plays an important role in innate immunity and the regulation of inflammation in the lung [5]. SP-D can activate intracellular phagocytosis and regulate the regeneration of intracellular reactive oxygen species and cytokines [6]. A previous study has demonstrated that levels of inflammatory cytokines IL-6 and TNF-α in SP-D knockout mice with radiation-induced lung injury were significantly higher than those in wild-type mice [6]. Moreover, in the same study, exogenous SP-D supplementation delivered through the airways reduced lung injury. We have shown in our recent studies that besides expression in the lung, SP-D is also expressed in several other organs such as the pancreas [7] and kidney [8]. We also demonstrated that extra-pulmonary SP-D plays an important role in the pathogenesis of infectious disease through its role in the regulation of inflammatory signaling pathways and apoptosis [7, 8]. In addition, we have also shown that in a CLP-induced sepsis model, SP-D knockout mice showed a higher degree of severity of kidney injury than wild-type mice [9].

The human SP-D gene is located in chromosome 10q22.2-q23.1, and contains many single nucleotide polymorphisms (SNP) [10]. Among these polymorphisms, three missense mutation loci in exons of SP-D gene have been described which result in alterations in codons corresponding to amino acid residues 11 (Met11Thr), 160 (Ala160Thr) and 270 (Ser270Thr) [10]. It has been reported previously that Met11Thr SNP is associated with the susceptibility to acute lung injury (ALI)/acute respiratory distress syndrome (ARDS) [11]. In this study, adult ALI/ARDS patients with SP-D 11Thr/Thr genotype had higher levels of SP-D correlated with an increase risk of mortality in patients with ALI/ARDS [12].

Given the association between SP-D-Met11Thr SNP and the susceptibility to ALI, and SP-D expression in renal tubular epithelial cells [8], we hypothesized that SP-D polymorphisms may be associated with susceptibility to AKI. Therefore, in the present prospective control study, Chinese AKI patients and healthy controls of a Han background were recruited to explore the associations SP-D polymorphisms Thr11Met and Thr160Ala and serum SP-D level with the severity and prognosis of AKI.

## Methods

### Subjects

This study included 159 AKI patients (88 female and 71 male) aged 18–60 years, who were admitted in the Department of Critical Care Medicine of Renmin Hospital Wuhan University,Wuhan, a central region of China, located in the Hubei Province, between March 2012 and June 2013; And 120 age-matched healthy volunteers (50 female and 70 male) with no acute or chronic diseases which were recruited as controls in the same hospital. All participants recruited in this study were Han Chinese. The diagnosis and stage of AKI were established in accordance with the criteria of Kidney Disease Improving Global Outcomes (KDIGO) issued by the International Society of Nephrology (ISN) in 2012 [13]. AKI was defined as serum creatinine (Scr) elevation exceeding 0.3 mg/dL (26.5 mol/L) within 48 h, or Scr elevation exceeding 1.5 fold of the baseline value, or urine output less than 0.5 mL/kg/h for more than 6 h. AKI was classified into three stages: Stage 1, Scr elevation exceeding 1.5–1.9 fold of the baseline level, or Scr elevation exceeding 0.3 mg/dL (26.5 mol/L) or urine output less than 0.5 ml/kg/h for 6–12 h; Stage 2, Scr elevation exceeding 2.0–2.9 fold of the baseline level, or urine output less than 0.5 mL/kg/h for more than 12 h; Stage 3, Scr elevation exceeding 3.0 fold of the baseline level, or Scr elevation exceeding 4.0 mg/dL (353.6 μmol/L), or need for renal replacement therapy (RRT). Patients younger than 18 years or older than 70 years of age, pregnant women, and patients with chronic renal disease and renal contusions were excluded from the study. The study protocol was approved by the Ethics Committee of our hospital, and written informed consent was obtained from all patients and subjects before initiation of the study.

### Specimen collection

Peripheral blood (5 mL) samples were collected from AKI patients and healthy controls, and stored at room temperature for 1 h, then centrifuged at 5000 rpm/min for 5 min to separate serum and white blood cells. Urine (10 mL/per patient) samples were collected in addition. Clinical data of AKI patients were recorded, including general demographic data, etiology of AKI, stage of AKI, Apache II score, and duration of RRT as well as prognosis of disease.

### Analysis of SP-D Thr11Met and Thr160Ala polymorphisms

For SNP analysis, genomic DNA was extracted from peripheral white blood cells of patients and healthy controls according to the instructions of the genome DNA extraction kit (Solarbio, Beijing, China). The final DNA concentration was between 0.06 and 0.12 μg/μL as detected by UV spectrophotometer. Using genomic DNA as the template, SP-D polymorphisms SP-D Thr11Met and Thr160Ala were examined by sequence specific primer-polymerase chain reaction (PCR-SSP) with appropriate primers as described previously [14]. This method provided the reproducible results for all the SNP loci with the PCR conditions as follows: initial denaturation 1 min at 94 °C; followed by 5 cycles of 20s at 94 °C, 45 s at 65 °C, 25 s at 72 °C; 21 cycles of 25 s at 94 °C, 50s at 55 °C, 30s at 72 °C; 4 cycles of 30s at 94 °C, 60s at 50 °C, 120 s at 72 °C; and final extension at 72 °C for 3 min. After amplification, PCR products were separated and identified using 2%-agarose gel electrophoresis.

### Measurement of serum SP-D protein and urine kidney injury molecule-1 (KIM-1) levels by ELISA

Serum SP-D protein and urine KIM-1 levels in AKI patients and healthy controls were detected by ELISA (R&D Inc, Minneapolis, United States) according to the manufacturer’s instructions.

### Statistical analysis

Statistical analysis was performed using SPSS software version 13.0 (SPSS Inc., Chicago, IL). Hardy-Weinberg equilibrium (HWE) was used to determine whether the genotype and allele frequencies were consistent with the genetic balance. Allele and genotype frequencies were compared by Pearson’s two-tailed chi-squared test or Fisher exact test (for sample number <5 in a group). Odds ratios with a 95% confidence interval were calculated using logistic regression analysis. Quantitative data were expressed as X ± SEM and compared by ordinary one-way ANOVA test or Student’s t- test where appropriate. Associations between serum SP-D levels and urine KIM-1 levels and AKI stage were analyzed by Pearson correlation analysis. A *P* value less than 0.05 was considered to be statistically significant.

## Results

### Clinicopathological characteristics of AKI patients and healthy controls

Clinicopathological characteristics of AKI patients and healthy controls included in our study are presented in Table 1. The average age of AKI patients and healthy controls was 45.09 ± 8.02 and 44.37 ± 7.64 years, respectively. The average Scr level of AKI patients was significantly higher than that of healthy controls (2.67 ± 1.02 mg/dL vs. 0.73 ± 0.17 mg/dL, *p* < 0.05). Among the 159 AKI patients, 57 (35.8%) developed KIDGO AKI stage 1, 64 (40.3%) developed KIDGO AKI stage 2, and 38 (23.9%) developed KIDGO AKI stage 3. From all AKI patients in 89 (56%) AKI was caused by sepsis, in 30 (18.9%) AKI was caused by trauma, in 25 (15.7%) AKI was caused by cardiovascular disease, and in 15 (9.4%) AKI was caused by other diseases. The average APACHE II score of the AKI patients was 20.3 ± 4.58. Out of 159 AKI patients, 60 (37.7%) AKI patients were treated with RRT, and the average RRT duration was 10.6 ± 3.38 days. Of the 60 AKI patients treated with RRT, 35 (58.3%) patients were treated with 25 mL/kg RRT dose, and 25 (41.7%) patients were treated with 35 mL/kg RRT dose.

**Table 1:** Clinicopathological characteristics of AKI patients and healthy controls

	AKI (*n* = 159)	Controls (*n* = 120)
Age (yr)	45.09 ± 8.02	44.37 ± 7.64
Male/Female	71/88	70/50
Creatinine (mg/dl)	2.67 ± 1.02	0.73 ± 0.17
Apache II score	20.3 ± 4.58	NA
AKI Stage		
Stage 1 (n,%)	57 (35.8)	NA
Stage 2 (n,%)	64 (40.3)	NA
Stage 3 (n,%)	38 (23.9)	NA
Mortality	49 (30.8)	NA
Cause of AKI		
Sepsis (n,%)	89 (56.0)	NA
Trauma (n,%)	30 (18.9)	NA
Cardiovascular (n,%)	25 (15.7)	NA
Other (n,%)	15 (9.4)	NA
RRT (n,%)	60 (37.7)	NA
RRT (days)	10.6 ± 3.38	NA
RRT Dose		
25 ml/kg (n,%)	35 (58.3)	NA
35 ml/kg (n,%)	25 (41.7)	NA

### Association between SP-D polymorphisms and susceptibility to AKI

The distribution of SP-D Thr11Met and Thr160Ala genotypes and alleles obtained in our study were similar to previous studies in the Chinese population, and were consistent with HWE (*p* > 0.05). Compared with healthy controls, the frequency of 11Thr/Thr genotype was significantly increased in AKI patients (*p* = 0.001). In addition, the frequency of 11Thr allele in AKI patients was also significantly higher than in healthy controls (*p* = 0.001). Moreover, the frequency of the 11Thr/Thr genotype was significantly higher in the subgroup of sepsis-induced AKI patients and patients who subsequently died when compared to the subgroup of the controls and survivors, respectively (*p* = 0.001& *p* = 0.0013), suggesting that SP-D-Thr11Met polymorphisms may be a predictor of worse outcomes in AKI patients. No significant differences in terms of genotype and allele frequencies at Thr160Ala loci were observed between AKI patients and healthy controls (*p* = 0.269) (Tables 2 and 3).

**Table 2:** Surfactant protein D gene polymorphisms in AKI patients and healthy controls

Genotype/allele	AKI patients, n(%)	Controls, n(%)	Odds ratio (95% CI)	*P* value	AKI patients		Odds ratio (95% CI)	*P* value
					Dead, n(%)	Alive, n(%)		
Met11Thr								
Met/Met	42 (26.4)	51 (42.5)	0.486 (0.293–0.805)	0.007**	9 (18.4)	43 (39.1)	0.351 (0.155–0.795)	0.011
Met/Thr	67 (42.1)	54 (45.0)			20 (40.8)	47 (42.7)		
Thr/Thr	50 (31.5)	15 (12.5)	3.211 (1.699–6.067)	0.001**	20 (40.8)	20 (18.2)	3.103 (1.468–6.557)	0.005**
Allele								
Met	151 (47.5)	156 (65.0)	0.487 (0.345–0.687)		38 (38.8)	133 (60.5)	0.414 (0.254–0.675)	
Thr	167 (52.5)	84 (35.0)	2.054 (1.455–2.899)	0.001**	60 (61.2)	87 (39.5)	2.414 (1.482–3.933)	0.0004**
Ala160Thr								
Ala/Ala	70 (44.0)	50 (41.7)	1.101 (0.682–1.778)	0.715	7 (14.3)	15 (13.6)	1.056 (0.401–2.779)	0.913
Ala/Thr	70 (44.0)	50 (41.7)			24 (49.0)	50 (45.5)		
Thr/Thr	19 (12.0)	20 (16.6)	0.679 (0.344–1.337)	0.297	18 (36.7)	45 (40.9)	0.839 (0.419–1.679)	0.619
Allele								
Ala	210 (66.0)	150 (62.5)	1.167 (0.823–1.655)		38 (38.8)	80 (36.4)	1.108 (0.679–1.810)	
Thr	108 (34.0)	90 (37.5)	0.857 (0.604–1.216)	0.422	60 (61.2)	140 (63.6)	0.902 (0.552–1.474)	0.707

*CI* confidence interval

**P values less than 0.01

**Table 3:** Surfactant protein D gene polymorphisms in sepsis-induced AKI patients and healthy controls

Genotype/allele	Sepsis-induced AKI patients, n(%)	Controls, n(%)	Odds ratio (95% CI)	*P* value
Met11Thr				
Met/Met	20 (22.5)	51 (42.5)	0.392 (0.212–0.726)	0.003**
Met/Thr	40 (44.9)	54 (45.0)		
Thr/Thr	29 (32.6)	15 (12.5)	3.383 (1.681–6.810)	0.001**
Allele				
Met	80 (44.9)	156 (65.0)	0.440 (0.296–0.654)	
Thr	98 (55.1)	84 (35.0)	2.275 (1.529–3.384)	0.001**
Ala160Thr				
Ala/Ala	35 (39.3)	49 (40.8)	0.952 (0.544–1.666)	0.888
Ala/Thr	43 (48.3)	52 (43.3)		
Thr/Thr	11 (12.4)	20 (15.9)	0.712 (0.322–1.574)	0.437
Allele				
Ala	113 (63.5)	150 (62.5)	1.043 (0.698–1.559)	
Thr	65 (36.5)	90 (37.5)	0.959 (0.642–1.433)	0.919

*CI* confidence interval

**P values less than 0.01

### Serum SP-D and urine KIM-1 levels in AKI patients and healthy controls

In our study SP-D serum level of healthy controls ranged from 56.21 ng/mL to 130.8 ng/mL (median, 80.36 ng/mL; 25–75^th^ IQR 74.27–88.68 ng/mL). Serum SP-D level at day 1 (baseline SP-D level) of AKI patients ranged from 110.6 ng/mL to 199.5 ng/mL (median, 142.4 ng/mL; IQR 128.5 ng/mL-158.5 ng/mL), from 275 ng/mL to 393.2 ng/mL at day 3 (median, 342.2 ng/mL; IQR 323.2 ng/mL-356.1 ng/mL) and from 100.4 ng/mL to 159.9 ng/mL at day 7 (median, 134.8 ng/mL; IQR 125.6 ng/mL-146.5 ng/mL). Compared with healthy controls, serum SP-D levels at day 1, 3 and 7 were significantly elevated (*p* < 0.01). Furthermore, SP-D serum levels at day 3 in AKI patients were significantly higher than that at day 1 and 7 (*p* < 0.01) (Table 4).

**Table 4:** Serum SP-D and Urine KIM-1 levels in AKI patients and healthy controls

	Median (IQR) serum SP-D (ng/ml)			Median (IQR) Urine KIM-1 (ng/ml)
Time	Day 1	Day 3	Day 7	Day 1
AKI patients, n(%)	142.4(128.5–158.5)^c^	342.2(323.2–356.1)^a^	134.8(125.6–146.5)^b^	1.37(1.14–1.58)^c^
Controls, n(%)	80.36(74.27–88.68)	NA	NA	0.47(0.23–0.64)

^a^Compared with Day 1,significant difference was observed. P less than 0.01

^b^Compared with Day 3, significant difference was observed. P less than 0.01

^c^Compared with healthy controls, significant difference was observed. P less than 0.01

Urine KIM-1 level in AKI patients was significantly higher than that in the healthy controls (*p* < 0.01) (Table 4) Urine KIM-1 level of healthy controls ranged from 0.23 ng/mL to 0.64 ng/mL (median, 0.47 ng/mL; 25–75^th^ IQR 0.39–0.53 ng/mL) while the urine KIM-1 level in AKI patients ranged from 0.89 ng/mL to 1.98 ng/mL (median, 1.37 ng/mL; IQR 1.14–1.58 ng/mL). The dynamic change of serum SP-D and urine KIM-1 levels were associated with the progression of AKI.

### Association between SP-D polymorphisms and serum SP-D level in AKI patients

In our study, AKI patients with the 11Thr/Thr genotype had significantly higher baseline (day 1) serum SP-D levels than those with either 11Met/Thr (*p* = 0.04) or 11Met/Met genotypes (*p* = 0.002). No difference however was observed in serum SP-D levels between AKI patients with 160Thr/Thr genotype and those with 160Ala/Thr and 160Ala/Ala genotypes (*p* = 0.12) (Table 5).

**Table 5:** Baseline serum SP-D levels and different genotypes among patients with AKI

AKI patients genotype	Median (25^th^-75^th^ IQR) baseline serum SP-D(ng/ml)	*P* value
Met11Thr		
Met/Met	123.8 (120.5–140.1)	
Met/Thr	135.4 (128.2–169.3)	0.040*
Thr/Thr	149.0 (144.5–158.8)	0.002**
Ala160Thr		
Ala/Ala	144.3 (121.6–169.9)	
Ala/Thr	138.3 (131.8–147.6)	0.091
Thr/Thr	144.4 (124.8–147.6)	0.150

*IQR* interquartile range

*P values less than 0.05; **P values less than 0.01

### Relationship between baseline serum SP-D level and urine KIM-1 level with AKI severity and need and duration of RRT in AKI patients

**Fig. 1:**
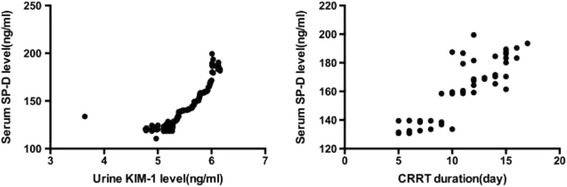
The correlation of baseline serum SP-D level with urine KIM-1 or CRRT duration in AKI patients

In our study, baseline serum SP-D levels (day 1) correlated with urine KIM-1 levels in AKI patients (Pearson coefficient, *r* = 0.658), indicating that higher levels of baseline serum SP-D, related to more severe renal injury (Fig. 1).

The baseline serum SP-D levels of AKI patients treated with RRT were also higher than in AKI patients without RRT (*p* = 0.012). In addition, baseline serum SP-D level correlated with the duration of RRT (Pearson co-efficient *r* = 0.852). However, there was no difference in the baseline serum SP-D levels between patients treated with different RRT doses (20 mL/kg vs. 35 mL/kg) (Table 6).

**Table 6:** Baseline serum SP-D levels and patients’ outcome, AKI stage and RRT of AKI patients

AKI patients	Outcome		AKI stage			RRT	
	Dead	Alive	Stage 1	Stage 2	Stage 3	With	Without
Median(25^th^ – 75^th^ IQR) serum SP-D(ng/ml)	149.6 (143.8–159.2)	139.5* (124.1–154.6)	124.3 (120.4–123.4)	137.7* (131.3–144.0)	156.1^##^(148.9–165.1)	142.7 (139.5–159.8)	140.3* (121.7–155.8)
*P* value	0.026		1	0.02	0.001	0.012	

For the serum SP-D levels and different outcome analysis

*Compared with AKI patients who died, significant difference was observed. P less than 0.05

For the serum SP-D levels and different AKI stage analysis

*Compared with AKI stage 1,significant difference was observed. P less than 0.05

^##^Compared with AKI stage 3,significant difference was observed. P less than 0.01

For the serum SP-D levels and RRT need

*Compared with AKI patients with RRT, significant difference was observed. P less than 0.05

## Discussion

The objective of this study was to explore the possible relationships between SP-D Thr11Met and Thr160Ala polymorphisms and serum SP-D levels as well as susceptibility, severity and prognosis of AKI in Chinese patients.

Previous studies have also demonstrated that the SP-D-Met11Thr polymorphism is associated with susceptibility to several diseases. For example, subjects from Western countries with 11Thr allele or 11Thr/Thr genotype were more susceptible to allergic rhinitis [15], asthma [16], chronic obstructive pulmonary disease (COPD) [17], and community-acquired pneumonia [18]. Interestingly, recipients of allografts with SP-D 11Met/Met genotype had significantly lower rates of chronic lung allograft dysfunction and improved survival compared to those with the homozygous SP-D 11Thr/Thr genotype [19]. In our study, individuals with 11Thr/Thr genotype were more susceptible to AKI compared to those with other SP-D genotypes. In addition, to avoid potential problems due to different race, region and living environments among subjects, all AKI patients and healthy controls in this study were of Chinese Han nationality and resided in similar geographic locations in the central area of China. In our study, frequencies of genotypes at Thr11Met and Thr160Ala loci in healthy controls were similar to those reported in previous studies conducted in the Chinese population [20]. However, the distribution of genotypes in the Chinese population differed from that reported in Western populations [11]. Thus, based on our findings, we can speculate that the SP-D-Thr11Met polymorphism may be used a biomarker to predict patient susceptibility to AKI of Chinese patients.

Among many SNPs present in the SP-D gene, two intraexonic polymorphisms, Met11Thr and Ala160Thr, which result in changes in amino acids residues, have been described. The Met11Thr polymorphism is located in codon 11 in the SP-D N-terminal region and it has been reported previously that this amino acid change influences the oligomerization of the human SP-D protein thereby impacting on its function [10]. Furthermore, SP-D protein posttranslational modifications such as nitrosylation of multimeric SP-D could cause collagen tail wrapping and affect SP-D binding ability to the calreticulin/CD91 receptor on macrophages. This, in turn, might lead to higher anti-inflammatory activity as compared with normal trimeric SP-D protein [21]. Moreover, a previous study demonstrated that multimeric SP-D protein was a better inhibitor of Gram-positive and negative bacteria as well as influenza virus A compared to SP-D, with a lower degree oligomerization [22]. In our study, we found that AKI subjects with 11Thr/Thr genotype had higher serum SP-D levels than those with 11Met/Met and 11Thr/Met genotypes, which may also contribute to individual susceptibility to AKI. These findings might be explained by the possibility that SP-D Thr11Met SNP could influence the assembly, expression, function and concentration of SP-D protein, consequently altering susceptibility to diseases in which SP-D is implicated.

A previous study by Eisner et al. [23] demonstrated that higher baseline plasma SP-D levels were associated with a greater risk of mortality in acute respiratory distress syndrome patients. Indeed, higher baseline serum SP-D levels were associated with worse clinical outcomes, including a higher degree of kidney injury, longer RRT duration and increased risk of death in our study. It has been shown previously that alveolar type II epithelial cell hyperplasia can be stimulated by intra-tracheal administration of KGF-2 to increase SP-D secretion. SP-D easily disseminates into blood vessels due to its hydrophilic nature, therefore, serum SP-D level may be used as a sensitive marker of the permeability of alveolar epithelial cells [24]. In the present study, serum SP-D levels in AKI patients were significantly higher than that in healthy controls, and reached a peak at day three. We speculate that increased serum SP-D levels in AKI patients may be attributed to large amounts of SP-D protein secreted by renal tubular epithelial cells which may accumulate in renal tubular lumen. In addition, SP-D protein from the tubular lumen may leak into the lumen of blood vessels when the barrier function of tubular epithelial cells is damaged. The permeability of tubular epithelial cells is related with the severity of renal injury, When the kidney suffers injury, and the more severely the renal epithelial cell barrier is damaged, the more SP-D protein would leak into blood from tubular lumen,which may explain the significant correlation between higher serum SP-D levels and higher stage of renal injury in AKI patients.

KIM-1 is a reliable and early predictor for AKI, as demonstrated by preclinical and clinical trials [25]. KIM-1 expression was most dominantly found in tubular epithelial cells, especially in S3 segment in ischemia/reperfusion injury (IRI) and toxic injury [25]. KIM-1 soluble fragment can also be detected in the urine of early AKI patients [26]. Urine KIM-1 levels have been recognized as a useful biomarker to occurrence and severity of sepsis-induced AKI [26]. In the present study, we found that urine KIM-1 levels in AKI stage 3 patients were significantly higher than that in AKI stage 2 and stage 1 patients. In addition, the baseline serum SP-D levels were significantly correlated with urine KIM-1 levels, which further suggested that serum SP-D levels may be a predictor for the severity and prognosis of AKI. Indeed, SP-D meets the criterion of a potentially ideal marker for AKI: it is non-invasive, easily obtainable and detectable early in patient samples.

Moreover, in the present study we observed that higher baseline SP-D serum levels were related to greater possibility of RRT and its longer duration. RRT is an effective way for AKI patients healing, which may be attributed to effective removal of inflammatory cytokines from the circulation and local renal tissue [27]. However, the mortality of AKI patients cannot be improved by high-dose RRT [28]. In our study no difference in serum SP-D levels at day three was observed among AKI patients who were treated with different doses of RRT (35 mL/kg vs. 25 mL/kg). Based on these findings, it may be that serum SP-D of AKI patients was not affected by different doses of RRT in our study.

## Conclusion

In conclusion, in our study, carriers of SP-D 11Thr allele/genotype were more susceptible to AKI compared to 11Met allele/genotype carriers. Furthermore, higher baseline serum SP-D levels were associated with adverse clinical outcomes, including higher mortality, greater possibility of RRT and longer duration. Therefore, the SP-D-11Thr allele/genotype and serum SP-D protein levels might perhaps be useful as biomarkers in predicting AKI susceptibility and prognosis. It was the first time to analyze the relationship between SP-D protein level and polymorphisms and susceptibility and outcome of AKI patients which can provide experimental evidences to compare the use of biomarkers in routine clinical practice. However, the sample number was not high which would be improved in the following study.

## Abbreviations

AKI: Acute kidney injury
ALI: Acute lung injury
ARDS: Acute respiratory distress syndrome
CLP: Cecal ligation and puncture
COPD: Chronic obstructive pulmonary disease
ELISA: Enzyme-linked immunosorbent assay
HWE: Hardy-Weinberg equilibrium
ICU: Intensive care unit
IL-6: Interleukin-6
IRI: Ischemia/reperfusion injury
KDIGO: Kidney Disease Improving Global Outcomes
KIM-1: Kidney injury molecule-1
PCR: Polymerase chain reaction
PCR-SSP: Sequence specific primer-polymerase chain reaction
RRT: Renal replacement therapy
SNP: Single nucleotide polymorphism
SP-D: Surfactant protein-D
TNF-*α*: Tumor necrosis factor-*α*
WBC: White blood cells
